# Phase I study of lapatinib plus vinorelbine in patients with locally advanced or metastatic breast cancer overexpressing HER2

**DOI:** 10.1038/bjc.2011.591

**Published:** 2012-01-12

**Authors:** E Brain, N Isambert, F Dalenc, V Diéras, J Bonneterre, K Rezai, M Jimenez, F Mefti-Lacheraf, E Cottura, P Tresca, L Vanlemmens, C Mahier-Aït Oukhatar, F Lokiec, P Fumoleau

**Affiliations:** 1 Department of Medical Oncology, Institut Curie – Hôpital René Huguenin 35 rue Daily, 92210 Saint-Cloud, France; 2 Department of Medical Oncology, Centre Georges-François Leclerc, 21034 Dijon, France; 3 Plurithematic Clinical Investigation Center, INSERM U803 Unit, 21034 Dijon, France; 4 Department of Medical Oncology, Institut Claudius Regaud, 31052 Toulouse, France; 5 Department of Medical Oncology, Institut Curie, 75231 Paris, France; 6 Department of General Cancerology, Centre Oscar Lambret, 59020 Lille, France; 7 Department of Pharmacology, Institut Curie – Hôpital René Huguenin, 92210 Saint-Cloud, France; 8 Department of Clinical and Therapeutic Trials, Fédération Nationale des Centres de Lutte Contre le Cancer, 75013 Paris, France

**Keywords:** anti-HER2 therapy, breast cancer, lapatinib, phase I study, vinorelbine

## Abstract

**Background:**

To determine the recommended doses of lapatinib (LPT) combined with vinorelbine (VNR) in women with human epidermal growth factor receptor 2-overexpressing advanced breast cancer pretreated with trastuzumab.

**Methods:**

In this phase I study, women were treated with oral daily LPT and i.v. VNR infused on days 1 and 8 every 3 weeks. Dose levels (DL) of LPT (mg)/VNR (mg m^−2^) ranged from 750/20 to 1250/30. The primary end point was feasibility based on maximal tolerated dose (MTD) and maximum administered dose (MAD). Pharmacokinetic interactions were investigated.

**Results:**

Of 33 patients included, 29 were evaluable. Two DLT occurred at DL4 (1000/25) meeting the MAD criteria. Despite an additional intermediate DL3′ (1250/22.5), MTD was reached at DL3 (1000/22.5). Grade 3–4 neutropenia was the most common toxicity (34% and 38% of patients, respectively). Other significant toxicities included grade 3–4 diarrhoea (3% each), and grade 3 asthenia (10%). Although not statistically significant, LPT (at 1000 or 1250 mg) decreased the VNR clearance by 30–40% compared with DL1.

**Conclusion:**

The MTD LPT 1000 mg/VNR 22.5 mg m^−2^ (DL3) is recommended for additional development. Pharmacokinetic interactions might increase the exposure to VNR and consequently alter the hematological tolerance.

Overexpression of human epidermal growth factor receptor-2 (HER2) is reported in 25–30% of metastatic breast cancers, conferring to these tumours a more severe prognosis (Dean-Colomb and Esteva, 2008). Conversely, patients with such tumour may benefit from specific targeted treatments. Trastuzumab is a humanised monoclonal antibody that binds to the domain IV of the extracellular segment of HER2 and has proven to be active in HER2-overexpressing breast tumours; the advantage of adding the monoclonal antibody to standard systemic treatments has been demonstrated in a meta analysis with consistent improvement in overall survival and progression-free survival (Harris *et al*, 2011). Yet drug resistance appears within 1 year in more than half of the patients receiving trastuzumab monotherapy. Although the combination with cytotoxic agents improves this outcome, approximately 15% of the patients still relapse (Nahta and Esteva, 2006) warranting the development of alternative treatments.

Lapatinib (LPT) is a potent orally active tyrosine kinase inhibitor, which blocks both epidermal growth factor receptor and HER2 (Rusnak *et al*, 2001). LPT binds to the cytoplasmic ATP-binding site of the kinase and blocks receptor phosphorylation and activation, thereby preventing subsequent downstream signaling events, namely, simultaneous activation of extracellular signal-related kinase-1/2 and phosphatidylinositol 3 kinase/Akt (Moy and Goss, 2006). The activation of mitogen-activated protein kinase is also inhibited in preclinical models (Xia *et al*, 2004). In addition, LPT induces apoptosis of HER2-overexpressing breast cancer cells resistant to trastuzumab (Nahta *et al*, 2007). This drug has been investigated in several studies, demonstrating significant efficacy and favourable tolerance profile in monotherapy after trastuzumab failure (Blackwell *et al*, 2004; Burstein *et al*, 2008). Limited adverse events are reported, mostly related to skin (rash) and gastrointestinal disorders (Blackwell *et al*, 2004). In a phase III study, the combination of LPT with capecitabine led to a significant increase in median time to progression and median progression-free survival compared with the antimetabolite alone (Geyer *et al*, 2006).

Vinorelbine (VNR) is a semisynthetic vinca alkaloid that inhibits cancer cell growth by acting on the mitotic cycle. An i.v. and oral formulations of VNR have been evaluated in combination with various cytotoxic agents in numerous studies for the treatment of metastatic breast cancer (Delozier *et al*, 2006; Sano *et al*, 2006; Savio *et al*, 2006). The drug has also proven efficacy and acceptable safety in association with trastuzumab (Burstein *et al*, 2001; Chan *et al*, 2006), overall response rates ranging from 44% to 86% (Chan, 2007). The main limiting toxicity of VNR is neutropenia. Other rare but significant (grade 3–4) side effects are alopecia, nausea and vomiting, neuropathy, paralytic ileus and localised dermal necrolysis at the site of i.v. injection.

These data provided the rationale for investigating the feasibility and the potential synergy of LPT combined with VNR in patients with HER2-positive disease progressing under trastuzumab. The 3-week schedule for VNR (day 1 and day 8 every 3 weeks) was chosen on the basis of current clinical practice in Europe. VNR being metabolised through cytochrome P450 3A4 (Kajita *et al*, 2000) and LPT being a strong mechanism-based inactivator of cytochrome P450 3A4 (Teng *et al*, 2010), pharmacokinetic interactions were also investigated.

## Materials and methods

### Eligibility criteria

Patients eligible to this phase-I dose-escalation multicentre trial were women >18-year-old with histologically confirmed HER2-overexpressing locally advanced or metastatic breast cancer. HER2 overexpression was defined by a 3+ immunohistochemistry score, or a 2+ score with positive fluorescence *in-situ* hybridisation. Patients were to have received one or two lines of chemotherapy with trastuzumab, in adjuvant or metastatic setting. Trastuzumab was to be stopped at least 3 weeks before study entry. Additional inclusion criteria were a WHO performance status ranging from 0 to 2, adequate hematological, hepatic, cardiac and renal functions. Ineligibility criteria included concomitant treatment by cytochrome P450 3A4 modulators (inhibitors or inducers) or pH modifying agents, significant gastrointestinal troubles affecting oral intake and prior treatment with VNR. All patients signed an informed consent.

### Study design, treatment, end points and dose escalation

Patients included received an oral loading dose of LPT for 7 days (day −7 to day 1 of cycle 1) in order to reach a steady state level for LPT before the first administration of VNR. Whereas the LPT intake was daily and continuous, VNR was administered i.v. by a 15-min infusion on day 1 and day 8 every 3 weeks. The eight pre-defined dose levels (DL) for LPT (mg)/VNR (mg m^−2^) were: 750/20, 1000/20, 1000/22.5, 1000/25, 1250/25, 1500/25, 1250/27.5 and 1250/30. Primary prophylaxis of neutropenia with granulocyte-colony stimulating factors was not permitted in cycle 1 and left to the investigator's choice from cycle 2.

The primary end point was the tolerance and feasibility based on (i) the maximal tolerated dose (MTD) defined as the highest DL tested with <2 dose-limiting toxicity (DLT), observed in a maximum of nine patients (in a 3+3+3 dose escalation design) and (ii) the maximum administered dose (MAD) defined as the highest DL tested with at least 2 DLT (⩾2) out of three to six patients.

DLT was defined on tolerance observed during cycle 1 only, as follows: grade 4 neutropenia lasting >7 days, grade 3–4 febrile neutropenia (>38.5°C), grade 4 or symptomatic grade 3 thrombocytopenia, omission or delay of day 8 of VNR owing to haematological toxicity, or any grade 3–4 non-haematological toxicity, excluding fatigue, anorexia, nausea and vomiting, and if considered clinically significant and drug-related by the investigator.

**Figure 1:**
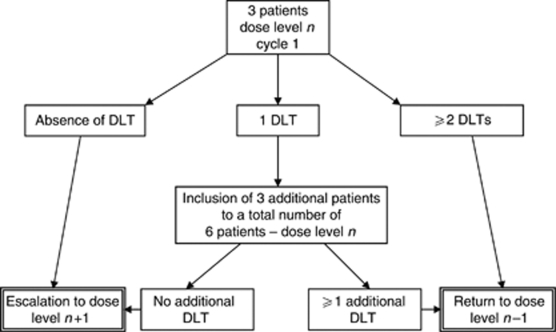
Study design. DLT=dose-limiting toxicity.

Three patients were initially planned at each DL. If no DLT was observed at DLn, enrollment could proceed at DLn+1 with three patients. In case of one DLT observed at DLn, three additional patients were to be included at the same DLn, allowing further escalation to DLn+1 only if no further DLT was observed (i.e. ⩽1 DLT in six patients). The occurrence of a second DLT at DLn met the criteria for MAD (⩾2 DLT in three or six patients) and MTD had to be further confirmed at DLn-1 with three to six additional patients, to make a total of nine patients in the cohort (Figure 1). There was no intra-patient dose escalation.

The study was expected to accrue a minimum of 12 and a maximum of 60 patients.

Treatment was pursued unless disease progression, significant toxicity or the patient's voluntary withdrawal occurred.

The study was approved by a central national ethics committee and the French National Drug Agency. The protocol was reviewed by the internal review board of all participating institutions. It was conducted in accordance with Good Clinical Practice guidelines and the Declaration of Helsinki.

### Assessments

As mentioned above, the primary end point of the study was tolerance and feasibility based on MTD and MAD defined according to DLT recorded during cycle 1. Only patients who completed the LPT loading dose period and at least day 1 of cycle 1 (i.e. first VNR infusion) were evaluable for the primary end point. Patients not assessable for DLT were to be replaced. All patients receiving at least one dose of the study drugs were included in the efficacy and general safety analyses. Toxicity was graded according to the National Cancer Institute Common Terminology Criteria for Adverse Events, version 3 (NCI CTCAE, v3.0, Bethesda, MD, USA; http://ctep.cancer.gov/protocolDevelopment/ electronic_applications/ctc.htm#ctc_30). Tumour response was evaluated every 2 cycles according to the RECIST criteria. Seven blood samples were collected for pharmacokinetic analyses on day 1 of cycle 1: before VNR infusion, then 12 and 30 min, 1, 3, 6, 12 and 24 h after VNR infusion. LPT and VNR concentrations were assessed by liquid chromatography coupled with tandem mass spectrometry (Van Heugen *et al*, 2001; Bai *et al*, 2006). Pharmacokinetic parameters were estimated by non-linear mixed effect modeling.

## Results

### Patients’ characteristics

From August 2007 to March 2010, 33 women were enrolled in five French centres. Patients’ characteristics are summarised in Table 1. Mean time between study entry and disease progression was 6±11.8 weeks. They had received 1 or 2 lines of trastuzumab-based treatment in 64% and 36% of cases, respectively. A minor protocol violation was reported in two patients who had received three lines of treatment that included trastuzumab.

### Primary end point

Of 33 patients enrolled, 29 patients were assessable for DLT analysis. One patient never started any treatment because of non-eligibility criteria and three others received only the loading dose of LPT before discontinuing. No DLT occurred at DL1, DL2 and DL3. Of three patients included at DL4 (1000/25), two developed an haematological DLT (one febrile neutropenia and one grade 4 neutropenia lasting >7 days) meeting the criteria for MAD. According to the initial design, DL3 (1000/22.5) had to be considered as the MTD and recommended for further development. However, preliminary DL1-DL4 pharmacokinetic data showed a trend for a negative correlation between LPT dose and VNR clearance (see below), warranting the exploration of an immediate higher and approved LPT dose with the VNR dose used in DL3 in order to confirm potential pharmacokinetic interactions and to decide whether full dose anti-HER2 therapy could be maintained in the final combination. This led to add an extra DL3′ (1250/22.5) to the original design. Of nine patients eventually enrolled at DL3′, two developed a DLT: one grade 4 neutropenia >7 days, and one grade 4 neutropenia and diarrhoea with confusion, multivisceral failure and pancreatitis (Table 2). Thus, DL3 (1000/22.5) was further expanded to a total of 11 patients and validated as the MTD with no DLT observed.

### General safety and observance

Of 33 enrolled patients, 32 received at least one dose of one study drug. These 32 patients totalised 226 cycles of treatment with a median number of 4 cycles (0–24).

The overall toxicity seen in the study is summarised in Table 3.

Significant toxicity occurring during cycle 1 encompassed grade 3–4 neutropenia (34% and 38% of patients, respectively), grade 3 and 4 diarrhoea (3% each) and grade 3 asthenia (10%). Throughout the study, grade 3–4 neutropenia occurred in 75% of patients. Grade 1–2 diarrhoea, rash and transaminitis were reported in 53%, 28% and 43.8% of patients, respectively. There was no grade 3–4 skin toxicity. One patient developed grade 2 dyspnea following VNR infusion. Death from toxicity and grade 3–4 cardiac events were not reported.

Excluding the 4 patients who did not receive any VNR, 23 patients discontinued from the study in relation to toxicity, disease progression or patient's choice (2, 18 and 3 cases, respectively). VNR and LPT doses had to be reduced in 14% and 6% of cycles, respectively. VNR administration was on an average delayed in 21.5% of all cycles: 14%, 27%, 16%, 30% and 23% in DL1, DL2, DL3, DL4 and DL3′, respectively.

### Pharmacokinetics

**Figure 2:**
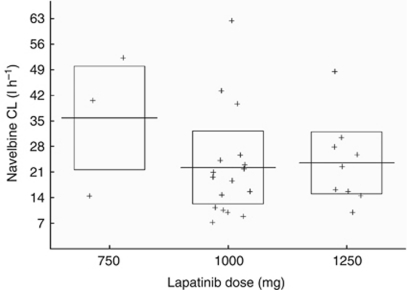
Vinorelbine clearance according to dose of lapatinib. CL=clearance.

The blood concentrations of VNR and LPT could be measured in 29 patients with 115 and 169 time points available for VNR and LPT, respectively. The mean population clearance value for VNR was 24.9 l h^−1^, lower than historical values (Zhou and Rahmani, 1992). It decreased inversely according to LPT doses, although this decrease did not reach statistical significance (Figure 2). Conversely, the LPT clearance was not affected by the VNR dose. According to the model that we developed to describe VNR pharmacokinetics, the LPT dose (considered either as a categorical or a continuous covariate), which would decrease VNR clearance by 50% was established as 1790 mg. No clear correlation was observed between individual pharmacokinetic data (VNR clearance) and the occurrence of a DLT at DL4 and DL3′. The final model for VNR pharmacokinetic did not retain the LPT effect, according to the Akaike and Bayesian information criteria allowing comparisons of log-likelihood, and to the between-subject variability values. Detailed pharmacokinetic modeling data have been published in a separate report (Rezai *et al*, 2011).

### Response rates and follow-up

Of 33 patients enrolled, 7 were not evaluable for treatment anti-tumour effect: 4 patients with no VNR infusion and 3 others having stopped treatment before first assessment of response (1 refusal, 1 major protocol violation and 1 toxicity). No complete response was observed. Partial response was recorded in 8 patients (31%) with a median duration of response of 128 days (41–192) and with distribution according to DL as follows: 2/3 (67%), 0/3 (0%), 1/9 (11%), 2/3 (67%) and 3/8 (38%) patients in DL1, DL2, DL3, DL4 and DL3′, respectively. A total of 14 patients (54%) had stable disease with a median duration of 92 days (38–210). In all, 13 patients died from disease progression with a median follow-up after treatment of 13 months (0–21). Six patients were still on treatment at the time of the present analysis.

## Discussion

Metastatic breast cancer still bears a high mortality burden, and new drugs or new associations are eagerly awaited to widen therapeutic options and to improve prognosis. The high activity of both LPT and VNR taken individually or in association to other systemic treatment supported the investigation of their combination for metastatic HER2-positive breast cancer patients, after trastuzumab-based treatment failure. This study has evaluated the tolerability and feasibility of a daily LPT and a 3-week VNR regimen given in this setting. The primary end point was the determination of MTD and MAD. Finding haematological toxicity as the main issue for DLT and based on an expansion of DL3 cohort to a total of 11 patients, we now recommend the following doses for further investigation: LPT 1000 mg daily per o.s. and VNR 22.5 mg m^−2^ on days 1 and 8 every 3 weeks.

Few data are available to compare and discuss these results. In monotherapy, LPT has been used up to the daily dose of 1500 mg in two phase II studies (Blackwell *et al*, 2004; Burstein *et al*, 2008). In a phase III study, it was combined with capecitabine at a daily dose of 1250 mg (Geyer *et al*, 2006). To our knowledge, no phase I study investigating the optimal dosing regimen for the combination of LPT with VNR has been published so far.

The results of a phase I study (LAP 104241) led in the United States combining LPT and VNR were presented only as a poster in 2009 (Chew *et al*, 2009). In this study, patients were randomly assigned to LPT given daily and VNR infused on day 1, day 8 and day 15 every 4 weeks (arm A) or to LPT on days 2–5, 9–12 and 16–25 with the same VNR regimen (arm B). The MTD reported was LPT 1500 mg and VNR 20 mg m^−2^ (arm A) or LPT 1500 mg and VNR 25 mg m^−2^ (arm B).

Why the higher doses of LPT could be reached in this study remains unconfirmed. There are a few potential explanations. First, dosing regimens were different with three VNR infusions spread over 28 days compared with two infusions on a 3-week basis in our study. The other study included patients with prostate and lung cancers in addition to breast cancers. Also, the definition of DLT did not encompass long-lasting grade 4 neutropenia as in our study. This DLT was responsible for two of the four observed DLT. Lastly, no pharmacokinetic analysis was presented in this other study precluding further generation of hypotheses.

Another Taiwanese phase I study was also presented as a poster to the 2010 San Antonio Breast Cancer Symposium, evaluating the LPT and VNR combination in metastatic breast cancer progressing after trastuzumab and taxane-based treatment (Lu *et al*, 2010). However, VNR was administered orally making the comparison with our results difficult. The authors recommend the combination of LPT 1000 mg daily and VNR 50 mg m^−2^ orally on days 1 and 8 every 3 weeks. According to published bioequivalence data, VNR 50 mg m^−2^ might approach an i.v. dose of 20 mg m^−2^ (Marty *et al*, 2001).

Two phase II studies have been initiated and should provide further information on the optimal dosing regimen for this combination: the LPT 11110 study intends to treat 60 patients in a single-arm design with doses of LPT 1500 mg daily and VNR 20 mg m^−2^ on days 1, 8 and 15 every 4 weeks (http://www.clinicaltrials.gov/ct2/show/NCT00709618). The VITAL study (LAP 112620) will combine LPT 1250 mg daily and VNR 20 mg m^−2^ on days 1 and 8 every 3 weeks (http://clinicaltrials.gov/ct2/show/NCT01013740).

Regarding general safety, our results are consistent with others. The most common grade 3–4 toxicity is neutropenia that occurred in 75% of our patients as compared with 42% and 72% in arms A and B, respectively, of the American phase I study (Chew *et al*, 2009). The most common grade 3–4 non-haematologic toxicity was diarrhoea in both studies, rates approaching 15% of patients. In the Taiwanese study, no grade 4 toxicity occurred, the most frequent adverse events were diarrhoea whereas grade 3 neutropenia was reported in only 2 out of 15 patients (Lu *et al*, 2010).

Combined with our results, these data show that the combination of VNR and LPT exhibits a manageable safety profile, most adverse events being mild-to-moderate. In our experience, they resulted in treatment discontinuation in only two cases.

Response rates are difficult to interpret in phase I studies where they can be only considered as complementary observational data looking for hints of activity. The global clinical benefit reported in our study compare favorably with those reported in the others: 31% partial response and 51% stable disease, *vs* 10% and 37% in the American study (Chew *et al*, 2009) and 33% of stable disease in the Asiatic study (Lu *et al*, 2010). In comparison, the phase III study using LPT and capecitabine reported an overall response rate of 22% (Geyer *et al*, 2006). However, caution remains the rule regarding conclusions on efficacy given the modest response rate observed at DL3 that we recommend for further development.

Finally, pharmacokinetic analyses showed that LPT administration might be responsible for a decrease of VNR clearance by 30–40%, potentially depending on the dose of LPT used. Although not statistically significant, this trend might be a key clue to understand the lower recommended dose reached in our study for both VNR and LPT, in addition to the conservative definition of DLT including long-lasting asymptomatic neutropenia. That the final model developed for VNR pharmacokinetic eventually did not retain the LPT effect as a significant covariate (Rezai *et al*, 2011) might be explained by the small number of patients per DL, especially for DL1 (three patients). As a strong cytochrome P450 3A4 inhibitor, LPT might be responsible for an unfavourable modulation of VNR with a lower clearance inducing higher myelosuppression. Taken together with our results on DLT, these pharmacokinetic data provide a strong hypothesis to warrant the use of a cautious lower dose of VNR when combined with LPT or of a dose decrement for both drugs, as described for other tyrosine kinase inhibitors (Scheffler *et al*, 2011). No other pharmacokinetic data are currently available, but the ongoing VITAL study is expected to provide further information regarding the clinical relevance of this interaction potential and inhibitory effects on the metabolising enzymes and transporters.

### Conclusion

The recommended doses of daily LPT combined with i.v. VNR given in a 3-weekly schedule on days 1 and 8 are 1000 mg and 22.5 mg m^−2^, respectively. This combination is generally well tolerated. With the limited value of efficacy data in phase I studies, it provides encouraging efficacy results. A pharmacokinetic interaction may result in an increased exposure to VNR and jeopardise the safety profile of the combination. This warrants the use of lower doses of both drugs than the standard when the drugs are used individually. The lower dose of LPT does not appear to alter the impact of LPT as an anti-HER2 therapy.

## Figures and Tables

**Table 1:** Baseline patients’ characteristics (*N*=33)

**Characteristics**	**Number of patients *N* (%)**
*Age (years)*	
Median (range)	58 (36–75)
Menopause at study entry	30 (90.9)
	
*WHO performance status*	
0	15 (46.9)
1	17 (53.1)
Missing	1 (3.0)
	
*Hormonal status*	
ER-positive and/or PgR-positive	13 (39.4)
ER-negative and PgR-negative	18 (54.5)
Missing	2 (6.1)
	
*HER2 status*	
IHC 3+	28 (84.8)
IHC 2+ and FISH positive	5 (15.2)
	
*Metastatic status*	
Number of sites: median (range)	2 (1; 7)
Liver/lung/both	18/13/7
Bone/bone only	14/1
Brain/brain only	5/1
	
*Previous treatments*	
Radiotherapy	28 (84.8)
Hormonotherapy	15 (45.5)
Trastuzumab 1 line	21 (63.6)
Trastuzumab 2 lines	12 (36.4)^a^
Median number of lines of chemotherapy (range)	2 (1; 5)

Abbreviations: ER=estrogen receptors; FISH=fluorescence *in situ* hybridisation; IHC=immunohistochemistry; PgR=progesterone receptors.

a Two patients had received three lines of trastuzumab before enrollment.

**Table 2:** Dose-limiting toxicity (CTCAE, v3.0) according to dose level

**Dose level**	**LPT (mg per day)**	**VNR (mg m^−2^) i.v. on day 1 and day 8**	***N* (%)**	**Dose-limiting toxicity**
1	750	20	3 (9.1)	
2	1000	20	5^a^ (15.2)	
3	1000	22.5	11 (33.3)	
4	1000	25	3 (9.1)	1 febrile neutropenia 1 grade 4 neutropenia >7 days
3′	1250	22.5	11^b^ (33.3)	1 grade 4 neutropenia >7 days 1 grade 4 neutropenia and diarrhoea with confusion, multivisceral failure and pancreatitis

Abbreviations: i.v.=intravenous; LPT=lapatinib; NCI CTCAE, v3.0=National Cancer Institute Common Terminology Criteria for Adverse Events version 3; VNR=vinorelbine.

a Two non-evaluable patients: brain metastasis requiring corticotherapy and gastric metastases interfering with treatment intake.

b Two non-evaluable patients: concomitant prohibited medication and brain metastases.

**Table 3:** Toxicity (NCI CTCAE, v3.0)

	**Cycles *N*=226**				**Patients *N*=32**			
	**Grade 1–2**		**Grade 3–4**		**Grade 1–2**		**Grade 3–4**	
**Adverse event**	***N***	**%**	***N***	**%**	***N***	**%**	***N***	**%**
Neutropenia	55.0	24.3	65.0	28.8	3.0	9.4	24.0	75.0
Febrile grade 3–4 neutropenia	na	na	1.0	0.4	na	na	1.0	3.1
Anaemia	138.0	61.1	2.0	0.9	22.0	68.8	2.0	6.3
Thrombocytopenia	5.0	2.2	1.0	0.4	4.0	12.5	1.0	3.1
Vomiting	23.0	10.2	0	0	14.0	43.8	0	0
Diarrhoea	67.0	29.6	5.0	2.2	17.0	53.1	5.0	15.6
Constipation	12.0	5.3	0	0	9.0	28.1	0	0
Mucositis	14.0	6.2	1.0	0.4	5.0	15.6	1.0	3.1
Myalgia	18.0	8.0	0	0	5.0	15.6	0	0
Rash	17.0	7.5	0	0	9.0	28.1	0	0
Asthenia	128.0	56.6	7.0	3.1	22.0	68.8	5.0	15.6
Transaminitis	58.0	25.7	4.0	1.8	14.0	43.8	3.0	9.4
Bilirubin	7.0	3.1	1.0	0.4	3.0	9.4	1.0	3.1

Abbreviation: NCI CTCAE, v3.0=National Cancer Institute Common Terminology Criteria for Adverse Events, version 3 (NCI CTCAE, v3.0).
